# Comparison of the Anti-inflammatory Properties of Two Nicotinic Acetylcholine Receptor Ligands, Phosphocholine and *p*CF3-diEPP

**DOI:** 10.3389/fncel.2022.779081

**Published:** 2022-03-31

**Authors:** Katrin Richter, Roger L. Papke, Clare Stokes, Danika C. Roy, Eduardo S. Espinosa, Philipp M. K. Wolf, Andreas Hecker, Juliane Liese, Vijay K. Singh, Winfried Padberg, Klaus-Dieter Schlüter, Marius Rohde, J. Michael McIntosh, Barbara J. Morley, Nicole A. Horenstein, Veronika Grau, Alain R. Simard

**Affiliations:** ^1^Department of General and Thoracic Surgery, Laboratory of Experimental Surgery, Justus-Liebig-University, German Center for Lung Research, Giessen, Germany; ^2^Department of Pharmacology and Therapeutics, University of Florida, Gainesville, FL, United States; ^3^Department of Chemistry and Biochemistry, Laurentian University, Sudbury, ON, Canada; ^4^Department of Biology, Laurentian University, Sudbury, ON, Canada; ^5^Department of Pediatric Hematology and Oncology, Justus-Liebig-University, Giessen, Germany; ^6^Institute of Physiology, Justus-Liebig-University Giessen, Giessen, Germany; ^7^Department of Biology, University of Utah, Salt Lake City, UT, United States; ^8^George E. Wahlen Veterans Affairs Medical Center, Salt Lake City, UT, United States; ^9^Department of Psychiatry, University of Utah, Salt Lake City, UT, United States; ^10^Center for Sensory Neuroscience, Boys Town National Research Hospital, Omaha, NE, United States; ^11^Department of Chemistry, University of Florida, Gainesville, FL, United States; ^12^Northern Ontario School of Medicine, Sudbury, ON, Canada

**Keywords:** CHRNA7, CHRNA9, CHRNA10, cytokines, inflammation, silent agonist, monocytes, macrophages

## Abstract

Activation of nicotinic acetylcholine receptors (nAChRs) expressed by innate immune cells can attenuate pro-inflammatory responses. Silent nAChR agonists, which down-modulate inflammation but have little or no ionotropic activity, are of outstanding clinical interest for the prevention and therapy of numerous inflammatory diseases. Here, we compare two silent nAChR agonists, phosphocholine, which is known to interact with nAChR subunits α7, α9, and α10, and *p*CF3-N,N-diethyl-*N*′-phenyl-piperazine (*p*CF3-diEPP), a previously identified α7 nAChR silent agonist, regarding their anti-inflammatory properties and their effects on ionotropic nAChR functions. The lipopolysaccharide (LPS)-induced release of interleukin (IL)-6 by primary murine macrophages was inhibited by *p*CF3-diEPP, while phosphocholine was ineffective presumably because of instability. In human whole blood cultures *p*CF3-diEPP inhibited the LPS-induced secretion of IL-6, TNF-α and IL-1β. The ATP-mediated release of IL-1β by LPS-primed human peripheral blood mononuclear leukocytes, monocytic THP-1 cells and THP-1-derived M1-like macrophages was reduced by both phosphocholine and femtomolar concentrations of *p*CF3-diEPP. These effects were sensitive to mecamylamine and to conopeptides RgIA4 and [V11L; V16D]ArIB, suggesting the involvement of nAChR subunits α7, α9 and/or α10. In two-electrode voltage-clamp measurements *p*CF3-diEPP functioned as a partial agonist and a strong desensitizer of classical human α9 and α9α10 nAChRs. Interestingly, *p*CF3-diEPP was more effective as an ionotropic agonist at these nAChRs than at α7 nAChR. In conclusion, phosphocholine and *p*CF3-diEPP are potent agonists at unconventional nAChRs expressed by monocytic and macrophage-like cells. *p*CF3-diEPP inhibits the LPS-induced release of pro-inflammatory cytokines, while phosphocholine is ineffective. However, both agonists signal *via* nAChR subunits α7, α9 and/or α10 to efficiently down-modulate the ATP-induced release of IL-1β. Compared to phosphocholine, *p*CF3-diEPP is expected to have better pharmacological properties. Thus, low concentrations of *p*CF3-diEPP may be a therapeutic option for the treatment of inflammatory diseases including trauma-induced sterile inflammation.

## Introduction

Homopentameric nicotinic acetylcholine (ACh) receptors (nAChRs) composed of subunits α7 or α9 as well as α9α10 heteropentamers were originally described as ligand-gated ion channels with a high permeability for Ca^2+^ ions (Katz et al., 2000; Verbitsky et al., 2000; Uteshev et al., 2002; Lipovsek et al., 2014). In the nervous system, α7 nAChRs are widely expressed, whereas nAChR subunits α9 and α10 are confined to the outer hair cells of the inner ear (Elgoyhen et al., 2001; Lustig et al., 2001; Skok, 2002; Gotti et al., 2006). In addition, nAChR subunits α7, α9 and α10 are expressed by immune cells and numerous other non-neuronal cells (Kawashima and Fujii, 2003, 2004; Galvis et al., 2006; Wessler and Kirkpatrick, 2008; Koval et al., 2011; Beckmann and Lips, 2013; Kummer and Krasteva-Christ, 2014; Kawashima et al., 2015; Fujii et al., 2017a,b). While for these non-neuronal cells no convincing ionotropic functions have been published, diverse metabotropic functions were discovered (Peng et al., 2004; De Jonge and Ulloa, 2007; Razani-Boroujerdi et al., 2007; Hecker et al., 2009, 2015; Mikulski et al., 2010; Papke, 2014; Richter et al., 2016, 2018; Valbuena and Lerma, 2016; Zakrzewicz et al., 2017; Grau et al., 2018).

In innate immunity, nAChRs and their agonists are mainly involved in an attenuation of inflammation (Borovikova et al., 2000; Kox et al., 2009; Tracey, 2009; Simard et al., 2013; St-Pierre et al., 2016; Fujii et al., 2017a; Pavlov et al., 2018; Godin et al., 2020). Damage- or pathogen-associated molecular patterns (DAMPs or PAMPs) are sensed by pattern recognition receptors such as Toll-like receptors and typically induce the expression of numerous pro-inflammatory mediators and the release of inflammasome-independent cytokines such as interleukin (IL)-6 or tumor necrosis factor (TNF)-α (Kawai and Akira, 2010). This process is attenuated by activation of nAChRs in monocytes and macrophages (Borovikova et al., 2000; Simard et al., 2013; Yue et al., 2015; St-Pierre et al., 2016; Godin et al., 2020), which is of tremendous clinical interest for the treatment of numerous inflammatory diseases. Treatment of experimental sepsis in mice with specific α7 nAChR agonists or with vagal stimulation, which induces the release of endogenous ACh, attenuated hallmarks of inflammation (Borovikova et al., 2000; Wang et al., 2004; Pavlov et al., 2007; Tracey, 2009).

IL-1β, a potent pro-inflammatory cytokine of innate immunity, is mainly produced by monocytes and macrophages (Broz and Dixit, 2016; Dinarello, 2018). It plays a central role in host defense against infections, but also in the pathogenesis of sepsis as well as in numerous auto-inflammatory and autoimmune diseases (Dinarello, 2018; Mantovani et al., 2019; Galozzi et al., 2021). In the light of these Janus-faced properties of IL-1β, it is reasonable that mechanisms have evolved that tightly control the production and release of IL-1 β. For instance, the biosynthesis of the inactive pro-from of IL-1β (pro-IL-1β) in response to the activation of pattern recognition receptors is also down-regulated by nAChR α7 activation (Borovikova et al., 2000). The release of mature IL-1β typically depends on a second danger signal such as extracellular ATP, an indicator of cytoplasm spilled from damaged cells (Gross et al., 2011; Rathinam et al., 2012; Rathinam and Fitzgerald, 2016; Bortolotti et al., 2018; Dinarello, 2018). When the ATP-sensitive and ionotropic P2X7 receptor is activated, the NLRP3 (NACHT, LRR and PYD domains-containing protein 3) inflammasome assembles and allows self-activation of caspase-1, which cleaves pro-IL-1β and enables its swift release (Gross et al., 2011; Rathinam et al., 2012; Cekic and Linden, 2016; Rathinam and Fitzgerald, 2016). In addition to ATP, numerous DAMPs and PAMPs can induce the release of IL-1β in a P2X7 receptor-independent way (Broz and Dixit, 2016). We discovered that activation of nAChRs with conventional agonists, such as ACh, choline or nicotine, specifically inhibits the ionotropic function of monocytic ATP receptors and, hence, efficiently attenuates the ATP-induced inflammasome-dependent maturation and release of IL-1β (Hecker et al., 2015; Richter et al., 2016; Zakrzewicz et al., 2017). In this context, phosphocholine (PC) and other molecules with a PC-head group function as nAChR agonists. The inhibitory function induced by PC depends on nAChR subunits α7, α9 and α10 and is independent of ionotropic nAChR functions (Hecker et al., 2015; Richter et al., 2016, 2018). At human α9 nAChR and α9α10 nAChR heterologously expressed by *Xenopus laevis* oocytes, however, PC did not induce ion-currents, but ion-current responses to choline were attenuated at human α9α10 nAChRs in the presence of PC (Richter et al., 2016; Zakrzewicz et al., 2017).

DAMPs, like endogenous Toll-like receptor agonists and ATP, released for instance upon accidental or surgical trauma, induce damage-mediated life-threatening “sterile” inflammation even in the absence of infectious agents (Manson et al., 2012; Bortolotti et al., 2018). Unconventional nAChR agonists are, thus, promising starting points for the development of therapeutics. They ideally control both, the gene expression of pro-inflammatory cytokines (e.g., IL-6, TNF-α, pro-IL-1β) and the ATP-mediated maturation and release of IL-1β. Although there are no experimental data, PC is expected to be unstable and it is unknown, if PC can inhibit the gene expression of cytokines. An alternative approach targeting nAChRs, shown to be effective in animal models of inflammatory pain or autoimmune disease (Quadri et al., 2017; Godin et al., 2020), relies on the use of a recently described group of synthetic silent agonists of nAChR α7 based on a N,N-diethyl-N′-phenylpiperazine (diEPP) scaffold. These silent agonists need to be tested if they also inhibit the ATP-induced secretion of IL-1β.

Here we characterize the control of cytokine release by mononuclear phagocytes by PC in comparison to *p*CF3-diEPP. As expected, PC turned out to be unstable and ineffective in controlling lipopolysaccharide (LPS)-induced cytokine release. In contrast, we provide evidence, that *p*CF3-diEPP not only reduces cytokine release in response to Toll-like receptor activation with LPS, but also functions as a potent inhibitor of ATP-induced IL-1β release. We further show for the first time, that *p*CF3-diEPP is a partial agonist and a strong desensitizer of classical ionotropic functions at heterologously expressed human α9 and α9α10 nAChRs.

## Materials and Methods

### Chemicals and Reagents

ACh chloride (Cat# A6625), dimethyl sulfoxide (DMSO, Cat# D2650), LPS (*E. coli* 011:B4, Cat# L2630), macrophage colony-stimulating factor (M-CSF, Cat# SRP3110), mecamylamine hydrochloride (Mec, Cat# M9020), phorbol 12-myristate 13-acetate (PMA, Cat# P1585), PC (Cat# P0378), recombinant mouse interferon-γ (INF-γ, Cat# IF005), sodium bicarbonate (Cat# R8758), 3-aminobenzoate methanesulfonate (MS-222, Cat# E10521) and all chemicals for saline Ringer’s buffer preparations were purchased from Merck (Darmstadt, Germany) or from Millipore Sigma (St. Louis MO, United States). LPS from *E. coli* O26:B6 was purchased from Merck (Cat# L2654) or eBioscience (Thermo Fisher Scientific, Waltham MA, United States; Cat# L500497693). Gibco penicillin-streptomycin and L-glutamine were purchased from Thermo Fisher Scientific. BzATP [2′(3′)-O-(4-benzoyl-benzoyl)ATP trieethylammonium salt] was purchased from Jena Bioscience (Jena, Germany), EDTA from bioWORLD (Dublin, OH, United States) and recombinant human INF-γ from R&D Systems (Minneapolis, MN, United States).

*p*CF3-diEPP was synthesized as previously published (Quadri et al., 2017) and dissolved in DMSO. When appropriate, control experiments were performed with the corresponding concentrations of DMSO without *p*CF3-diEPP. The conopeptides [V11L, V16D]ArIB (specifically antagonizing α7 nACh (Whiteaker et al., 2008; Hone et al., 2009, 2010) and RgIA4 (antagonizing nAChRs composed of subunits α9 and α10 (Christensen et al., 2017; Romero et al., 2017; Grau et al., 2018) were produced and characterized as described previously (Innocent et al., 2008; Romero et al., 2017).

### Mouse Bone Marrow-Derived Macrophages

Male and female mice gene-deficient in *Chrna*7 (α7), *Chrna*9 (α9) or *Chrna*9/*Chrna*10 (α9/α10) as well as corresponding wild-type (WT) mice were used for isolation of bone marrow cells. The α9 (Chrna9tm1Bjmy MGI:5787807) and α10 (Chrna10tm1Bjmy MGI:5787808) knockout lines were generated by Genoway, Inc. (Lyon, France) and backcrossed to congenicity at the Boys Town National Research Hospital, Omaha, NE, United States (BTNRH) using C57Bl/6J mice from The Jackson Laboratory (JAX). The α7 animals were originally received from JAX and a colony maintained at BTNRH. The α7 animals were crossed with the α9 or α9/α10 to produce double and triple knockout lines. The animals used in the studies reported here were derived by crossing the double and triple knockout lines with C57Bl/6J mice to produce single α7 and α9 KOs and double α9/α10 KOs. This was done to produce backgrounds in the strains that were comparable. The use of the animals in the experiments reported here was approved by the BTNRH Institutional Animal Care Committee (protocol #16-04). Mice were bred and housed at BTNRH and shipped to the Laurentian University (Sudbury, Canada). Experimental animals received humane care according to NIH “Guide for the Care and Use of Laboratory Animals.” Animal experiments were reviewed and approved by the Laurentian University Animal Care Committee (file number 6013816). Genotypes were analyzed by PCR as described previously (St-Pierre et al., 2016; Morley et al., 2017).

Bone marrow cells were flushed from cleaned femoral and tibial bones using a 10 ml syringe and a 26–27-gauged needle. Debris was removed by passing the suspension through a 40 μm BD Falcon Cell Strainer (Becton, Dickinson (BD), Franklin Lakes, NJ, United States). Red blood cells were lysed by RBC Lysis buffer, as per the supplier’s protocol (BioLegend, San Diego, CA, United States). Cells were pelleted at 510 g for 10 min and 1 × 10^6^ cells/ml were resuspended in complete medium [RPMI 1640 medium (Cytiva, Marlborough, MA, United States) supplemented with 10% fetal calf serum (FCS; Life Technologies, Thermo Fisher Scientific, Cat# LS12484028), 50 U penicillin/ml, 50 μg streptomycin/ml and 2 mM L-glutamine] and 10 ng/ml M-CSF. 1 × 10^6^ cells per well were seeded in Costar 12-well plates (Corning, NY, United States) and cultured for 72 h. Thereafter, 1 ml of fresh complete medium was added supplemented with a final concentration of 10 ng/ml M-CSF, 10 ng/ml IFN-γ and 10 pg/ml LPS (*E. coli* O26:B6) to induce their differentiation into M1 macrophages. Cells were cultured for another period of 48 h. Then, the bone marrow-derived macrophages (BMDMs) were stimulated with higher concentrations of LPS (100 ng/ml) as described previously (Godin et al., 2020) in the absence and presence of PC (100 μM) or *p*CF3-diEPP (100 μM). Cell-free supernatant was harvested 6 h later and stored at −20°C for cytokine measurements.

### Human Whole Blood

For whole blood experiments, blood samples were obtained from healthy (self-reported) female and male non-smoking adult volunteers. Experiments were reviewed, approved, and performed in accordance with the policies outlined by the Laurentian University Research Ethics Board for Research Involving Human Participants (file number 6012214) and in accordance with the Helsinki Declaration. To prevent blood clotting, blood samples were collected in BD Vacutainer glass blood collection tubes containing sodium heparin (BD, Cat# B366489) and were individually shaken periodically during and after the blood draw. 200 μl of whole blood was added per well in Costar 24-well flat-bottom plates (Stemcell, Vancouver, Canada, Cat# 38017). Whole blood was then diluted in 700–800 μl of Sigma RPMI 1640 medium (Merck, Cat #R8758). Wells containing whole blood diluted in RPMI were pre-treated with 100 μM *p*CF3-diEPP or 0.1% DMSO for 1 h at 37°C. Immediately following pre-treatment, wells were stimulated with 100 ng/ml LPS (*E. coli* O26:B6) for 24 h (Bruchfeld et al., 2010). Controls wells were treated with an equivalent volume of Sigma RPMI 1640 medium. Following the 24 h of stimulation with LPS, cells were spun down (2,000 g, 5 min, RT). The cell-free supernatants were collected and stored at −20°C for later cytokine measurements.

### THP-1 Cells

Monocytic THP-1 cells (German Collection of Microorganisms and Cell Cultures, Braunschweig, Germany) were cultured in medium (Gibco*™* RPMI 1640 medium (Thermo Fisher Scientific, Cat# 11530586) supplemented with 10% FCS (CellConcepts, Umkirch, Germany) and 2 mM L-glutamine). To investigate IL-1β release, monocytic cells were resuspended in FCS-free RPMI medium and transferred to 48-well plates (Greiner Bio-One, Frickenhausen, Germany; 0.5 × 10^6^ cells/0.5 ml and per well). Cells were primed for 5 h with 1 μg/ml LPS (*E. coli* O26:B6) as described previously (Hecker et al., 2015). After priming, the P2X7 receptor agonist BzATP (100 μM) was added for 40 min in presence or absence of different concentrations of cholinergic agonists (PC, *p*CF3-diEPP) and antagonists (mecamylamine, [V11L, V16D]ArIB and RgIA4). After cell treatment, cells were spun down (500 g, 8 min, 4°C), the supernatants were collected and stored at −20°C for later cytokine measurements.

In addition, THP-1 cells were differentiated into M1-like macrophages. Monocytic THP-1 cells were resuspended in complete medium supplemented with 50 U penicillin/ml and 50 μg streptomycin/ml and seeded in 12-well plates (Greiner; 0.3 × 10^6^ cells/ml and per well). Cells were differentiated into macrophages by 24 h incubation with 50 nM PMA followed by 24 h incubation in complete medium supplemented with 50 U penicillin/ml, 50 μg streptomycin/ml. Macrophages were then polarized to M1-like macrophages by incubation in fresh complete medium, supplemented with 50 U penicillin/ml, 50 μg streptomycin/ml, 10 ng/ml recombinant human IFN-γ and 10 ng/ml LPS (*E. coli* 011:B4) for 72 h. To investigate IL-1β release, the medium was replaced by fresh complete medium and the cells were stimulated with LPS (*E. coli* O26:B6) and BzATP as described for monocytic THP-1 cells.

### Human Peripheral Blood Mononuclear Cells

Peripheral Blood Mononuclear Cells (PBMCs) were obtained from healthy (self-reported) female and male non-smoking adult volunteers. The study was approved by the ethics committee of the medical faculty Giessen, Germany (No. 90/18) and performed in accordance with the Helsinki Declaration. Blood was drawn into sterile syringes containing EDTA (1 mM per ml blood; bioWORLD, Dublin, OH, United States). LPS (*E. coli* O26:B6; 5 ng/ml) was added to blood samples shortly before PBMC isolation and PBMCs were separated using Leucosep gradients (Greiner) as described previously (Hecker et al., 2015). Thereafter, PBMCs were cultured for 3 h in 24-well plates (Greiner) at a density of 0.5 × 10^6^ cells/0.5 ml in Monocyte Attachment Medium (PromoCell, Heidelberg, Germany). Non-adherent cells were removed, and fresh Sigma RPMI 1640 medium was added. Stimulation with BzATP in the presence or absence of *p*CF3-diEPP was done as described for THP-1 cells. After cell treatment, cells were spun down (500 g, 8 min, 4°C), the supernatants were collected and stored at −20°C.

### Cytokine Measurements

In samples obtained in the mouse BMDM experiments, Mouse IL-6 Flex Set (BD, Cat# 558301), Mouse TNF Flex Set (BD, Cat# 558299), Mouse IL-10 Flex Set (BD, Cat# 558300) and Mouse IL-1β Flex Set (BD, Cat# 560232) were used to measure cytokine concentrations with the BD Cytometric Bead Array technique per the supplier’s protocol. Human IL-1β Flex kit (BD, Cat# 558279), Human IL-6 Flex kit (BD, Cat# 558276), Human IL-10 Flex kit (BD, Cat# 558274), and Human TNF Flex kit (BD, Cat# 560112) were used to measure cytokine concentrations in the supernatants of human whole blood experiments according to the manufacturer’s protocol. The bead fluorescence in the samples was quantified by flow cytometry using a BD FACS Canto II flow cytometer in conjunction with BD FACS Diva Software (v6.1.3). Cytokine concentrations were interpreted using BD FCAP Array Software (v3.0).

IL-1β concentrations in supernatants obtained in experiments on THP-1 cells (monocytic, M1-like) and human PBMCs were measured using the Human IL-1 beta/IL-1F2 DuoSet enzyme-linked immunosorbent assay (ELISA) from R&D Systems. To estimate cell viability at the end of the cell culture experiments on human PBMCs, the non-radioactive cytotoxicity assay (Promega, Madison, WI, United States) was used to measure lactate dehydrogenase (LDH) activity in cell-free supernatants as indicated by the supplier. LDH values are given as percentage of the total LDH content of lysed control cells.

### Expression of Human nAChR Subunits in *Xenopus laevis* Oocytes

Plasmid DNA encoding the human *CHRNA7* was obtained from Dr. J. Lindstrom (University of Pennsylvania, Philadelphia, PA, United States). The human resistance-to-cholinesterase 3 (*RIC3*) clone was obtained from Dr. M. Treinin (Hebrew University, Jerusalem, Israel) and co-injected with *CHRNA7* to improve the level and speed of receptor expression without affecting their pharmacological properties (Halevi et al., 2003). Plasmid DNA encoding the human *CHRNA9* and *CHRNA10* as well as the human 43 kDa receptor-associated protein of the synapse (*RAPSN*) with codon optimization for expression in *Xenopus laevis* were obtained from Eurofins Genomics (Ebersberg, Germany), (Richter et al., 2016). After linearization and purification of the plasmid DNAs, RNAs were prepared using the mMessage mMachine *in vitro* RNA transcription kit (Ambion, Austin, TX, United States).

Frogs were maintained in the Animal Care Service facility of the University of Florida, and all procedures were approved by the University of Florida Institutional Animal Care and Use Committee (approval number 202002669). In brief, the animals were first anesthetized for 15–20 min in 1.5 l frog tank water containing 1 g of MS-222 buffered with sodium bicarbonate. Oocytes were obtained surgically from mature female *Xenopus laevis* (Nasco, Ft. Atkinson, WI, United States) and treated with 1.4 mg/ml type 1 collagenase (Worthington Biochemicals, Freehold, NJ, United States) for 2–4 h at RT in Ca^2+^-free Barth’s solution (88 mM NaCl, 1 mM KCl, 2.38 mM NaHCO_3_, 0.82 mM MgSO_4_, 15 mM HEPES, and 12 mg/l tetracycline, pH 7.6) to remove the ovarian tissue and the follicular layers. Stage V oocytes were injected with 4–6 ng *CHRNA7* RNA and 2–3 ng *RIC3* RNA (2:1 ratio) in 50 nl water, or with 12 ng *CHRNA9* RNA and 3 ng *RAPSN* RNA, or along with 12 ng *CHRNA10* RNA in 50 nl water. Oocytes were maintained in Barth’s solution containing 0.32 mM Ca(NO_3_)_2_ and 0.41 mM CaCl_2_, and recordings were carried out 2–20 days after injection.

### Two-Electrode Voltage-Clamp Electrophysiology

Two-electrode voltage-clamp experiments were conducted using OpusXpress 6000A (Molecular Devices, Union City, CA, United States) (Papke and Stokes, 2010). Both the voltage and current electrodes were filled with 3 M KCl. Oocytes were voltage-clamped at −60 mV at RT. The oocytes were perfused with Ringer’s solution (115 mM NaCl, 2.5 mM KCl, 1.8 mM CaCl_2_, 10 mM HEPES, 1 μM atropine, pH 7.2) at 2 ml/min. To evaluate the effects of experimental compounds, responses were compared to control ACh-evoked responses, defined as the average of two initial applications of 60 μM ACh made before test applications. Drug applications were 12 s in duration followed by 181 s washout periods. In some experiments after obtaining the control responses, the bath perfusion solution was switched to the alternative buffer B, and test solutions delivered during the buffer B perfusion were made up in buffer B solution.

The responses were calculated as both peak-current amplitudes and net charge, as previously described (Papke and Porter Papke, 2002). Data were collected at 50 Hz, filtered at 20 Hz, and analyzed by Clampfit (Molecular Devices) and Excel (Microsoft, Redmond, WA, United States). Data were expressed as means ± SEM from at least four oocytes for each experiment and plotted with Kaleidagraph 4.5.2 (Abelbeck Software, Reading, PA, United States). Each episode of data acquisition was a total of 210 s and included an initial 30 s period used to define the baseline for the drug-evoked responses. After 30 s, drugs were applied, and the following 120 s were defined as the drug response period for analysis. Data reported for α7 were net charged, while peak currents were used for α9 and α9/α10 responses since these receptors do not show the same concentration dependent desensitization that invalidates peak currents as measurements of α7 concentration-dependent responses (Papke and Porter Papke, 2002). Multi-cell averages were calculated for comparisons of complex responses. Averages of the normalized data were calculated for each of the 10,322 points in each of the 206.44 s traces (acquired at 50 Hz), as well as the standard errors for those averages.

### Statistical Analyses and Data Processing

Results obtained in the LPS- and BzATP-induced cytokine release experiments were analyzed using SPSS (Version 23, IBM, Armonk, NY, United States). The IC_50_ value of *p*CF3-diEPP in human PBMCs was determined in GraphPad Prism (Version 6, GraphPad Software, San Diego, CA, United States) by fitting log-transformed concentration values and the original effect data. Paired data were analyzed first by the Friedman test followed by the Wilcoxon signed-rank test.

In two-electrode voltage-clamp experiments, the comparisons of results were made using one-way ANOVA or using *t*-tests between the pairs of experimental measurements. In cases, where multiple comparisons were made, a Bonferroni correction for multiple comparisons (Aickin and Gensler, 1996) was applied to correct for possible false positives. A value of *p* ≤ 0.05 was used to constitute a minimum level of significance. The statistics were calculated using an Excel template provided in Microsoft Office or ANOVA protocols in Kaleidagraph (4.5.2 Abelbeck Software, Reading, PA). Concentration-response relationships utilized data obtained over a range of concentration at roughly half log units. Average responses were fit to the Hill equation by the Levenberg-Marquardt algorithm in Kaleidagraph.

The number (n) of individual experiments is indicated in the Results section and the Figures. When primary cells were investigated, the n-number represents data obtained from the cells of individual mice or humans. In experiments with cell lines, the n-number refers to independent experiments, which were performed on different days with different cell passages. No outliers were excluded from the analyses. All two-electrode voltage-clamp experiments began with 8 oocytes voltage clamped and treated in parallel. Only cells, which lost voltage-clamp or otherwise failed to remain viable through the measurements, were excluded from the analyses but no viable cells. Data were visualized using Inkscape version 0.48.5 r10040 (Free and Open Source Software licensed under the GPL).

## Results

### Control of LPS-Induced Cytokine Release by pCF3-diEPP but Not by PC

**FIGURE 1:**
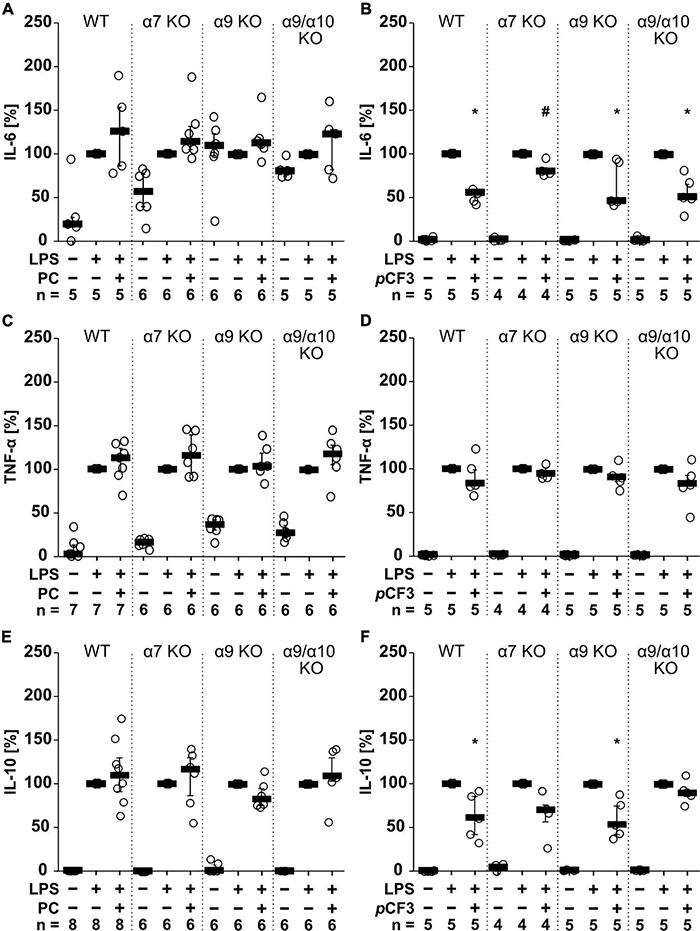
The effect of phosphocholine (PC) and *p*CF3-diEPP (*p*CF3) on lipopolysaccharide (LPS)-mediated cytokine release by mouse bone marrow-derived macrophages (BMDMs). BMDMs were isolated and cultured from wild-type (WT) and mice gene-deficient in *Chrna*7 (α7 KO), *Chrna*9 (α9 KO) or *Chrna*9 and *Chrna*10 (α9/α10 KO). On day 6 of cultivation, BMDMs were primed with lipopolysaccharide (LPS; 1 μg/ml; 5 h) in the absence and presence of PC (100 μM) or *p*CF3 (100 μM). The LPS-induced release of **(A,B)** interleukin-6 (IL-6), **(C,D)** tumor necrosis factor-α (TNF-α) and **(E,F)** IL-10 were measured in cell free cell culture supernatants by BD Cytometric Bead Arrays. Cytokine levels obtained after stimulation with LPS alone, were set to 100%, and all other values were calculated accordingly. Data are presented as individual data points, bars represent median, whiskers encompass the 25^th^ to 75^th^ percentile. *p ≤ 0.05, different from cells stimulated with LPS alone, Friedman-test followed by the Wilcoxon signed-rank test. #*p* ≤ 0.05, significantly different from the same condition in WT, Kruskal–Wallis followed by Mann–Whitney rank sum test.

Agonists of nAChRs have been shown to inhibit the production and release of pro-inflammatory cytokines by mononuclear phagocytes (Kox et al., 2009). Here, we investigated in M1-like murine BMDMs whether the unconventional nAChR agonist PC (Hecker et al., 2015; Richter et al., 2016) and the α7 silent agonist *p*CF3-diEPP (Quadri et al., 2017) change the levels of IL-1β, IL-6, TNF-α and IL-10 in cell culture supernatants in response to treatment with LPS (1 μg/ml for 5 h; Figure 1). Throughout, IL-1β levels remained below the level of detection. LPS induced the release of IL-6 in the range of 1 to 1,092 pg/ml, TNF-α in the range of 1,012 to 12,672 pg/ml and IL-10 in the range of 13 to 561 pg/ml. The LPS-induced release of these cytokines was not affected in the presence or absence of PC (100 μM; Figures 1A,C,E). In contrast, *p*CF3-diEPP (100 μM) significantly reduced IL-6 (Figure 1B; *p* = 0.007; *n* = 5) and IL-10 concentrations (Figure 1F; *p* = 0.007; *n* = 5) but no effect was seen on TNF-α (Figure 1D). In BMDMs from mice deficient in nAChR subunits α7, α9 or α10 as well as in mice with a double-deficiency in nAChR subunits α9 and α10, essentially the same results were obtained (Figure 1). Only the *p*CF3-diEPP-mediated attenuation of the secretion of IL-6 was not seen in BMDMs from nAChR α7 gene-deficient mice (Figure 1). These results are in line with previous reports that *p*CF3-diEPP functions as a silent agonist at nAChR α7 (Quadri et al., 2017). The fact that PC does not inhibit the LPS-induced production and secretion of cytokines, is most probably due to its expected instability, which is further confirmed in experiments described below (Supplementary Figure 1). Therefore, PC was omitted from the experiments on human whole blood cells.

**FIGURE 2:**
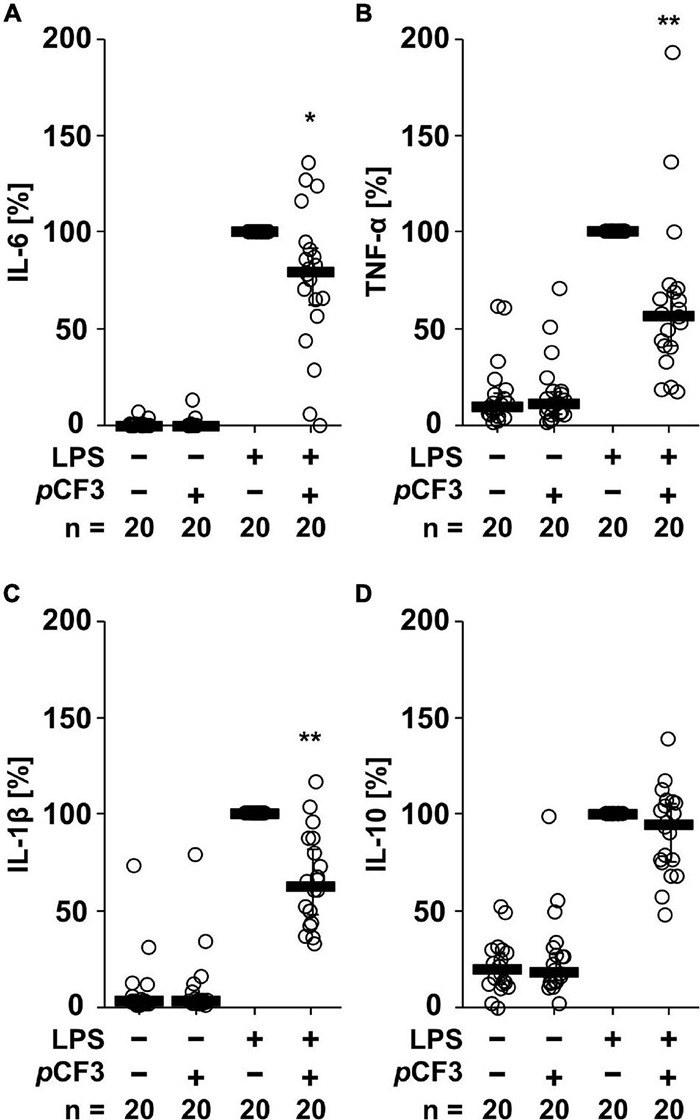
*p*CF3-diEPP (*p*CF3) inhibits the lipopolysaccharide (LPS)-induced release of interleukin-6 (IL-6), tumor necrosis factor-α (TNF-α) and IL-1β by human whole blood cells. Human whole blood was left untreated or pre-treated with 100 μM *p*CF3 for 1 h, followed by a 24 h stimulation with 100 ng/ml LPS. Thereafter, LPS-induced cytokine release was measured in cell-free cell culture supernatants by BD Cytometric Bead Arrays. In the presence of *p*CF3 the LPS-induced release of **(A)** IL-6, **(B)** TNF-α and **(C)** IL-1β was blunted, whereas no effect was seen on **(D)** IL-10. Cytokine levels obtained after stimulation with LPS alone were set to 100%, and all other values were calculated accordingly. Data are presented as individual data points, bars represent median, whiskers encompass the 25^th^ to 75^th^ percentile. *p ≤ 0.05 and **p ≤ 0.01 different from cells stimulated with LPS alone. Friedman-test followed by the Wilcoxon signed-rank test.

Human whole blood cells secreted IL-6 (in the range of 3 to 22,000 pg/ml), TNF-α (1 to 2,000 pg/ml), IL-1β (in the range of 3 to 5,600 pg/ml) and IL-10 (3 to 800 pg/ml), in response to LPS (100 ng/ml for 24 h; Figure 2). The LPS-induced release of IL-6 (*p* = 0.020), TNF-α (*p* = 0.003) and IL-1β (*p* = 0.0002) was significantly inhibited by *p*CF3-diEPP (Figures 2A–C), whereas no such effect was seen on IL-10 (*p* = 0.117; Figure 2D). In control experiments, in which LPS was omitted, *p*CF3-diEPP did not change the cytokine levels compared to supernatants of untreated cells (Figure 2).

### Control of the BzATP-Induced IL-1β Release by Human Monocytic Cells and Macrophages by PC and *p*CF3-diEPP

**FIGURE 3:**
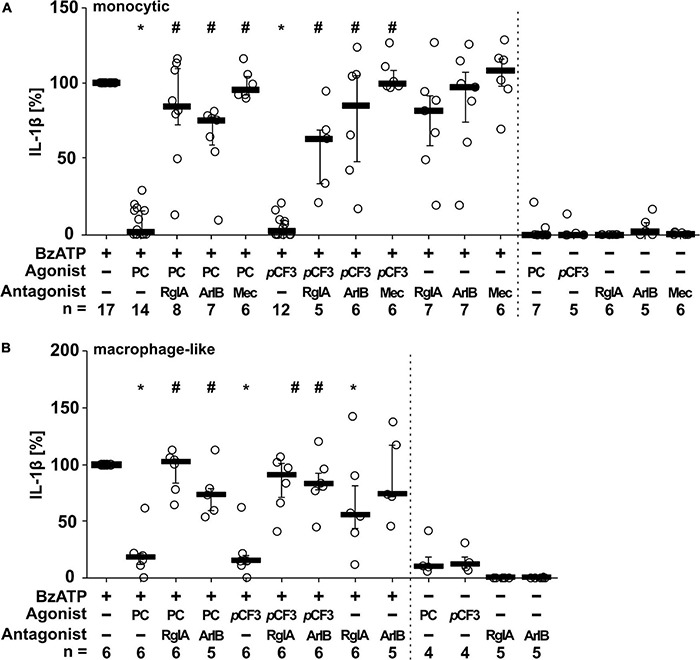
Phosphocholine (PC) and the silent agonist *p*CF3-diEPP (*p*CF3) inhibit the BzATP-mediated release of interleukin-1β (IL-1β) by monocytic and macrophage-like THP-1 cells *via* nAChR subunits α7, α9 and/or α10. Monocytic **(A)** and differentiated macrophage-like **(B)** THP-1 cells were used. Cells were primed with lipopolysaccharide (LPS; 1 μg/ml, 5 h). Thereafter, the P2X7 receptor agonist BzATP [2′/3′-O-(4-benzoylbenzoyl)adenosine-5′-triphosphate, tri(triethylammonium) salt] was added for another 40 min to trigger IL-1β release, which was measured by ELISA. The BzATP (100 μM)-induced release of IL-1β was investigated in the presence and absence of PC (200 μM), the synthetic silent agonists *p*CF3 (100 μM), antagonistic conopeptides RgIA4 (RgIA; 200 nM; specific for nAChRs composed of subunits α9 and α10) or [V11L, V16D]ArIB (500 nM; specific for α7 nAChR subunit) and the non-competitive, non-specific nAChR antagonist mecamylamine (Mec; 100 μM). The concentration of IL-1β released in response to BzATP was calculated by subtracting the IL-1β concentrations measured in supernatants of cells treated with LPS alone. In each experiment, the IL-1β concentration obtained after stimulation with BzATP was set to 100% and all other values were calculated accordingly. Data are presented as individual data points, bars represent median, whiskers encompass the 25^th^ to 75^th^ percentile. *p ≤ 0.05, different from LPS-primed cells stimulated with BzATP alone; ^#^*p* ≤ 0.05 significantly different from samples, where BzATP plus agonist was given. Friedman-test followed by the Wilcoxon signed-rank test.

We showed before that PC functions as an unconventional agonist at monocytic nAChRs containing subunits α7, α9 and α10 and as a potent inhibitor of the BzATP-induced maturation of IL-1β in monocytic U937 cells and human as well as murine peripheral mononuclear blood leukocytes (Hecker et al., 2015; Richter et al., 2016). To test if PC and *p*CF3-diEPP exert similar effects on monocytic THP-1 cells (Figure 3A) and THP-1-derived M1 macrophages (Figure 3B), cells were primed with LPS (1 μg/ml) for 5 h, followed by stimulation with the P2X7 receptor agonist BzATP (100 μM) for another 40 min in the presence or absence of PC (200 μM) or *p*CF3-diEPP (100 μM). Thereafter, the concentration of IL-1β was measured in cell culture supernatants by ELISA. As expected, untreated cells and cells primed with LPS did not release relevant amounts of IL-1β, whereas stimulation with BzATP resulted in elevated IL-1β levels released by monocytic THP-1 cells in the range of 19 to 122 pg/ml and by THP-1-derived M1-macrophages in the range of 53 to 314 pg/ml. In both monocytic cells and macrophage-like cells, PC and *p*CF3-diEPP significantly inhibited the BzATP-induced release of IL-1β. To examine the subtype(s) of nAChRs that underlie this effect, we used selective peptide antagonists beside the non-selective nAChR antagonist mecamylamine (Innocent et al., 2008). Homomeric α7, and α9-containing nAChRs can be difficult to pharmacologically differentiate. α-Bungarotoxin, for example, blocks both α7 and α9-containing nAChRs with low nM potency (Johnson et al., 1995; Briggs and McKenna, 1996; Elgoyhen et al., 2001). However, certain analogs of conotoxins have been developed that can effectively distinguish between these receptor subtypes (Grau et al., 2018). The inhibitory effect of PC and *p*CF3-diEPP on the BzATP-induced release of IL-1β was completely reverted by mecamylamine and blunted by the nAChRα7-specific conopeptide (Innocent et al., 2008; Whiteaker et al., 2008). [V11L, V16D]ArIB (500 nM) and by the α9α10 nAChR-specific conopeptide RgIA4 (200 nM), suggesting that nAChR subunits α7, α9 and/or α10 are involved in signaling (Figure 3A). In control experiments, in which mecamylamine, RgIA4 and [V11L, V16D]ArIB were given together with BzATP, the release of IL-1β was not significantly changed in monocytic THP-1 cells (Figure 3A). In THP-1-derived macrophage-like cells, however, a slightly reduced IL-1β concentration was measured in the presence of RgIA4, but not in the presence of [V11L, V16D]ArIB (Figure 3B). Further, PC, *p*CF3-diEPP, RgIA4, and [V11L, V16D]ArIB did not induce a relevant release of IL-1β in the absence of BzATP (Figures 3A,B).

To investigate the stability of PC and *p*CF3-diEPP in cell culture, PC (200 μM) and *p*CF3-diEPP (100 μM) were added to LPS-primed monocytic THP-1 cells 5, 15, and 30 min before stimulation with BzATP (100 μM). As shown in Supplementary Figure 1, the inhibitory effect of PC on the release of IL-1β was lost within 15 min, whereas *p*CF3-diEPP was effective throughout (Supplementary Figure 1).

**FIGURE 4:**
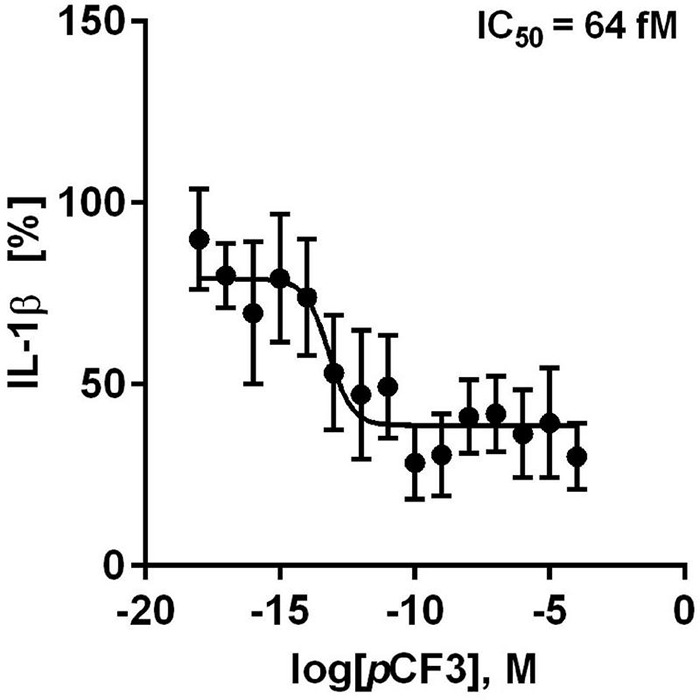
*p*CF3-diEPP (*p*CF3) inhibits the BzATP-mediated release of interleukin-1β (IL-1β) by human peripheral blood mononuclear cells (PBMCs). PBMCs were freshly isolated from blood of healthy human volunteers and primed with lipopolysaccharide (LPS, 5 ng/ml) during the isolation process. After 3 h of culture, non-adherent cells were decanted. *p*CF3 dose-dependently inhibited the BzATP [2’/3’-O-(4-benzoylbenzoyl)adenosine-5’-triphosphate, tri(triethylammonium) salt] (100 μM)-induced release of IL-1β by human PBMCs (IC_50_ = 64 fM). The amount of IL-1β released in response to BzATP was calculated by subtracting the IL-1β concentrations measured in supernatants of cells treated with LPS alone. In each experiment, the IL-1β concentration obtained after stimulation with BzATP was set to 100% and all other values were calculated accordingly. IL-1β (%) is provided on vertical axis and log molar (M) concentrations on horizontal axis. The IC_50_ value obtained by the dose-response curve (*n* = 5–9 independent experiments, shown as mean ± SEM) was obtained by non-linear regression analysis using Graph Pad Prism 6.0.

We further tested if *p*CF3-diEPP inhibits the BzATP-induced release of IL-1β by freshly isolated, LPS-primed human PBMCs. These cells released IL-1β in response to BzATP (in the range of 499 to 1,931 pg/ml), which was concentration-dependently inhibited by *p*CF3-diEPP (Figure 4). Of note, the calculated half maximal inhibitory concentration (IC_50_) value was as low as 64 fM (Figure 4). When different concentrations of *p*CF3-diEPP were added to LPS-primed PBMCs in control experiments without BzATP, IL-1β secretion remained at low levels (Supplementary Figure 2A). Cell viability as measured by LDH-release in LPS-primed and BzATP-stimulated PBMCs was not altered by different concentrations of *p*CF3-diEPP (Supplementary Figure 2B).

### Interference of PC and *p*CF3-diEPP With ACh-Induced Ion-Currents at Human α7, α9 and α9α10 nAChRs

**FIGURE 5:**
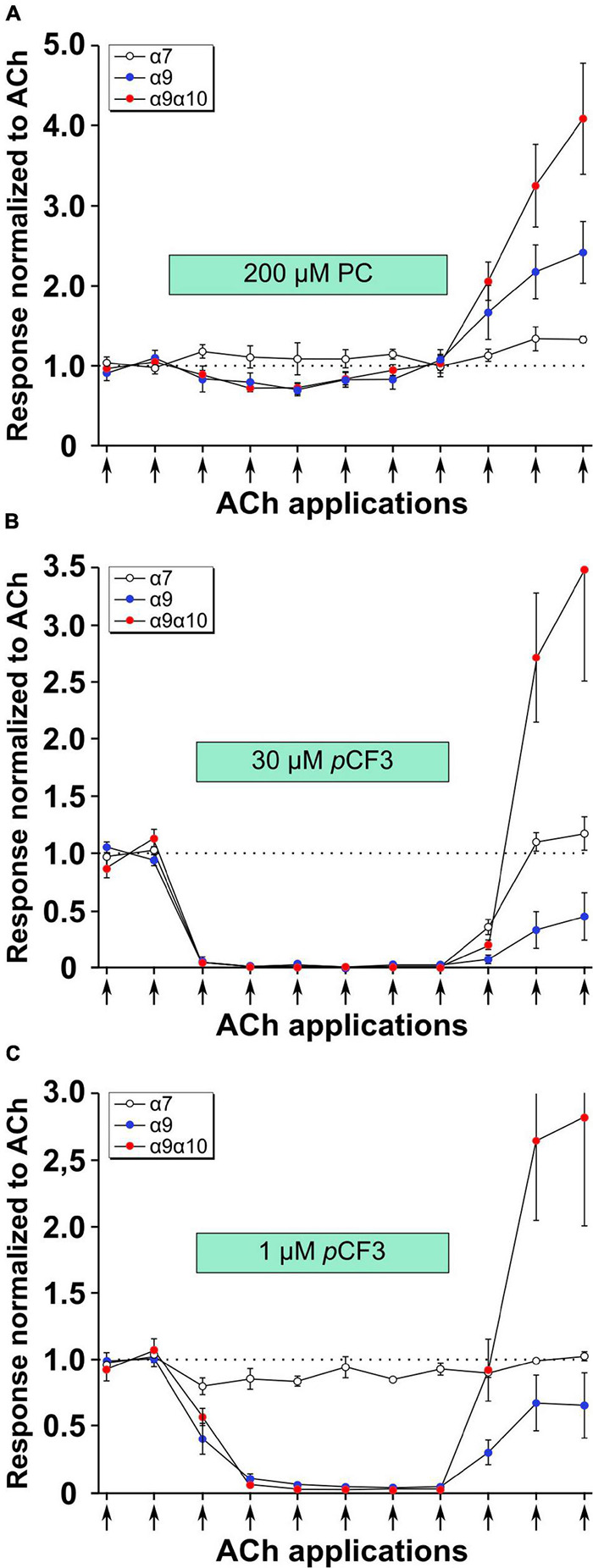
Effects of phosphocholine (PC) and *p*CF3-diEPP (*p*CF3) on the peak current responses evoked by acetylcholine (ACh) on *Xenopus laevis* oocytes expressing human α7, α9 or α9α10 nAChRs. Two-electrode voltage-clamp measurements were performed on *Xenopus oocytes* heterologously expressing α7, α9 or α9α10 nAChRs. Two initial current responses to 60 μM ACh (12 s pulses indicated by arrows) were obtained in control Ringer’s solution. Thereafter, the perfusion was switched to a solution containing **(A)** 200 μM PC, **(B)** 30 μM *p*CF3 or **(C)** 1 μM *p*CF3. The ACh-gated currents were monitored for changes in amplitude for six ACh applications. Thereafter, the perfusion solution was switched back to control and three more ACh-gated currents were monitored. Data are the average responses (± SEM), normalized to the average of the two initial ACh controls for each cell. The *n* values were 8, 6, and 7 for α7, α9 and α9 α10, respectively.

Previous experiments using 1 mM PC revealed an efficient PC-mediated reduction of choline-gated ion-currents at heterologously expressed α9α10 nAChRs (Richter et al., 2016). This prompted us to investigate the effects of PC and *p*CF3-diEPP on ACh-induced ion-currents in *Xenopus laevis* oocytes expressing human α7, α9 or α9α10 nAChRs in two-electrode voltage-clamp experiments. When ACh pulses (60 μM, 12 s, 2 ml/min) were applied to nAChR-expressing oocytes, repeatable ion-currents were induced (Figure 5). Concomitant application of PC (200 μM) had no effect at α7 nAChRs but provoked a slight reduction of the peak currents measured in oocytes expressing α9 or α9α10 nAChRs (Figure 5A). The concentration of 200 μM for PC was selected based on previous experiments (Richter et al., 2020) and on its potency to inhibit ATP-induced release of IL-1β as described above (Figures 3, 4). After wash-out of PC, ACh-induced peak currents increased most prominently in oocytes expressing α9α10 nAChRs, and to a lesser extent in oocytes expressing α9 or α7 nAChRs (Figure 5A). Of note, the ACh-induced ion currents in oocytes expressing α7, α9 or α9α10 nAChRs entirely vanished in the presence of 30 μM *p*CF3-diEPP (Figure 5B). When a concentration of 1 μM *p*CF3-diEPP was used in the same experimental setting, a minor attenuation of the ACh-induced peak currents was seen in oocytes expressing α7 nAChRs, whereas this concentration still fully inhibited ACh-gated currents at α9 or α9α10 nAChRs (Figure 5C). Interestingly, after wash-out of *p*CF3-diEPP, strongly increased peak currents induced by ACh were measured in oocytes expressing α9α10 nAChRs (Figures 5B,C). In contrast, such effects were not seen in oocytes expressing α7 nAChRs (Figures 5B,C). In oocytes expressing α9 nAChRs the responses to ACh after wash-out of *p*CF3-diEPP were reduced (Figures 5B,C).

**FIGURE 6:**
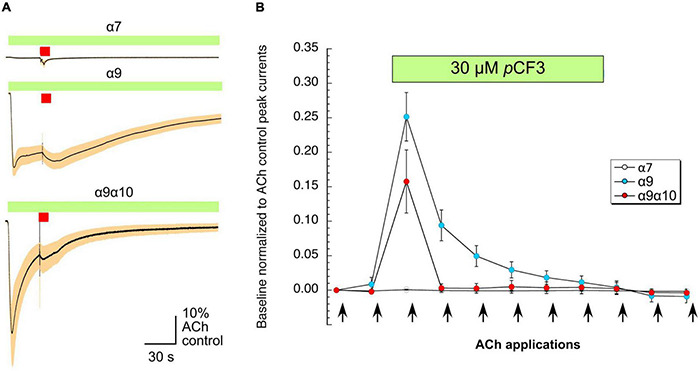
Baseline currents during the applications of 30 μM *p*CF3-diEPP (*p*CF3). Two-electrode voltage-clamp measurements were performed on *Xenopus laevis* oocytes heterologously expressing human α7, α9 or α9α10 nAChRs. **(A)** Averaged responses of oocytes heterologously expressing human α7 nAChRs (upper trace, *n* = 6), α9 nAChRs (middle trace, *n* = 7), or α9α10 nAChRs (lower trace, *n* = 7) to the onset of the bath application of 30 μM *p*CF3 and the co-application with acetylcholine (ACh, 60 μM, red bar). The data from each cell were first normalized to the peak current amplitude of the ACh control response that preceded the bath application of 30 μM *p*CF3. The normalized data were then averaged (black lines) at each of the 10,000 points in the data traces and SEMs calculated and displayed as the tan-colored bands. The scale bar represents 10% of the averaged ACh controls, which were 5.1 ± 1.3 μA, 128 ± 33 9 nA, and 2.95 ± 0.97 μA for α7, α9, and α9α10 nAChR-expressing cells, respectively. **(B)** The shift in baseline was measured over the initial 30 s intervals of subsequent acquisition periods as the absolute value of the average current during that time minus the holding currents prior to the first two ACh (12 s pulses, indicated by arrows) controls. These values were then normalized to the peak currents of the ACh controls in the same cells.

As shown in Figure 6A, the application of 30 μM *p*CF3-diEPP to the bath solution initiated a current response in the α7, α9 and α9α10 nAChR-expressing cells during the initial 30 s periods normally used to define the baseline for drug-evoked responses (see section “Materials and Methods”). *p*CF3-diEPP has previously been characterized as a very weak partial agonist for α7 nAChRs effective at inducing large responses only when co-applied with a positive allosteric modulator such as PNU-120596 (Quadri et al., 2017). Therefore, not surprisingly, there was minimal response of the α7-expressing cells, when the bath was switched to a solution containing 30 μM *p*CF3-diEPP (Figure 6A, upper trace). However, we noted substantial responses to the cells expressing α9 alone or α9α10 nAChRs (Figure 6A, middle and lower traces), suggesting that *p*CF3-diEPP is an activator of these receptors. The ACh applications during the period of *p*CF3-diEPP perfusion were diminished and, at least initially, superimposed on an increased baseline current activated by *p*CF3-diEPP (Figure 6B). For the cells expressing α9 nAChRs alone, this shift in baseline, apparently representing a steady-state activation of the receptors, did not decline fully during the 210 s period of data acquisition (Figures 6A,B). These data indicate that the steady-state current of the α9 nAChR-expressing cells remained elevated through several of the acquisition periods that followed the switch to the bath containing 30 μM *p*CF3-diEPP.

### Ion-Current Stimulation by *p*CF3-diEPP at Human α9 and α9α10 nAChRs

**FIGURE 7:**
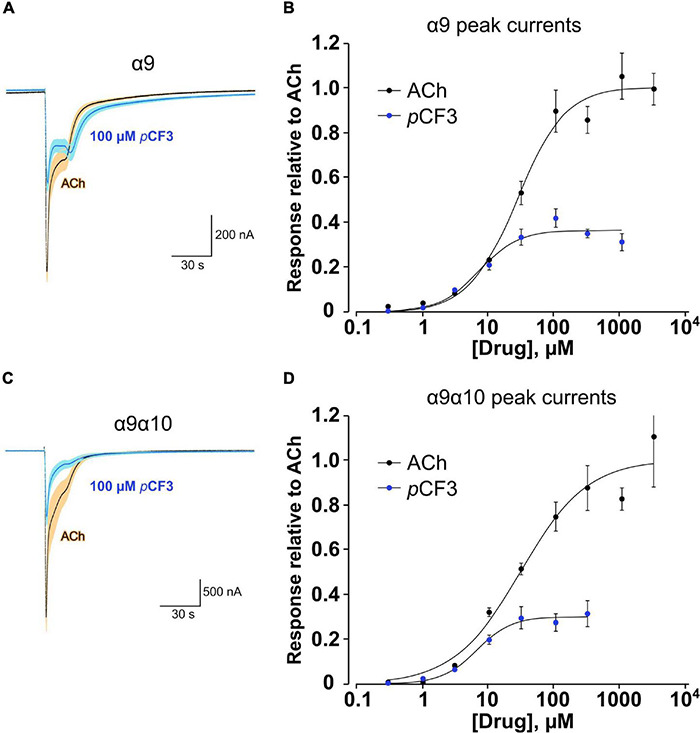
Concentration-response studies for acetylcholine (ACh) and *p*CF3-diEPP (*p*CF3) on heterologously expressed human nAChR subunits α9 and α9α10. Two-electrode voltage-clamp measurements were performed on *Xenopus laevis* oocytes heterologously expressing human α9 or α9α10 nAChR. **(A,C)** Averaged responses of oocytes expressing α9 nAChR (A, *n* = 6) or nAChR α9α10 [**(C)** *n* = 5] to 60 μM ACh in the absence and the presence of 100 μM *p*CF3. Individual cell responses were scaled to the peak amplitude of the ACh controls, then averaged (dark blue line) and plotted with the calculated SEM at each point (light blue band). These are compared to the averaged responses (black lines) and SEMs (tan bands) of the ACh control responses. **(B)** Average peak-current responses to ACh, normalized to the ACh maximum response of cells expressing subunit α9, compared to responses to *p*CF3 (*n* = 7). The *p*CF3 currents were initially measured relative to 60 μM ACh controls obtained prior to the *p*CF3 applications and then adjusted for the ratio of the ACh controls to ACh maximum by multiplying the values by 0.63. With the ACh I_Max_ = 1, the EC_50_ was 26.1 ± 4.2 μM (Chisq = 0.017, *R* = 0.9942). For *p*CF3, the I_Max_ was 0.36 ± 0.02 and the EC_50_ was 7.0 ± 1.9 μM (Chisq = 0.007, *R* = 0.979). **(D)** Similar experiments as described in **(B)** were performed on oocytes heterologously expressing α9α10 nAChRs. With the ACh I_Max_ = 1, the EC_50_ was 29.7 ± 9.9 μM (Chisq = 0.037, *R* = 0.986). For *p*CF3, the I_Max_ was 0.30 ± 0.01 and EC_50_ was 6.4 ± 0.9 μM (Chisq = 0.001, *R* = 0.995). For all concentration-response studies, the applications of the test compounds (ACh or *p*CF3) at progressively higher concentrations were alternated by repeated application of ACh at the control concentration of 60 μM to confirm that there was no rundown of the ACh controls through the course of the experiment.

We demonstrated before in *Xenopus laevis* oocytes heterologously expressing human α9α10 nAChR, that application of PC does not provoke ion-currents (Richter et al., 2016). Similarly, *p*CF3-diEPP functions as a very weak partial agonist in *Xenopus laevis* oocytes heterologously expressing α7 nAChRs (Quadri et al., 2017). Here, we performed two-electrode voltage-clamp measurements in *Xenopus laevis* oocytes heterologously expressing either human α9 nAChR or α9α10 nAChR. Of note, co-expression of α10 with α9 in the oocyte will favor the heteromeric composition of functional receptors compared to α9 alone (Elgoyhen et al., 2001; Sgard et al., 2002). Concentration-response studies of ACh or *p*CF3-diEPP were performed and the ion-current responses were normalized to those provoked by 60 μM ACh. ACh and *p*CF3-diEPP concentration-dependently stimulated ion-currents at α9 nAChRs (Figures 7A,B). The maximal ACh-induced ion-current (I_max_) was set to 1 and for *p*CF3-diEPP a relative I_max_ of 0.36 ± 0.02 was determined. The half maximal effective concentration (EC_50_) of ACh was 26.1 ± 4.2 μM, whereas that of *p*CF3-diEPP was 7.0 ± 1.9 μM. Similar ion-currents were induced at α9α10 nAChRs, with a relative I_max_ of 0.30 ± 0.01 for *p*CF3-diEPP, an EC_50_ for ACh of 29.7 ± 9.9 μM and an EC_50_ of 6.4 ± 0.9 μM for *p*CF3-diEPP (Figures 7C,D).

## Discussion

We demonstrate that the silent α7 nAChR agonist *p*CF3-diEPP inhibits the expression of pro-inflammatory cytokines by monocytes/macrophages in response to stimulation with the Toll-like receptor agonist LPS, while PC is ineffective. We further provide evidence that *p*CF3-diEPP, similar to PC, efficiently controls the maturation and release of monocytic IL-1β in response to P2X7 receptor stimulation, a mechanism that is mediated by nAChR subunits α7 and α9/α10. Surprisingly, *p*CF3-diEPP functions as a partial agonist and induces ionotropic functions at heterologously expressed human α9 and α9α10 nAChRs when given alone, while efficiently inhibiting ACh-gated ion currents.

Most reports on α7 nAChR-mediated anti-inflammatory effects describe an inhibition of the release of LPS-induced pro-inflammatory cytokines such as IL-6 and TNF-α (Borovikova et al., 2000; Kox et al., 2009; Simard et al., 2013; St-Pierre et al., 2016). Here, we show that PC did not change the LPS-induced cytokine release by murine M1-like BMDMs. In contrast, *p*CF3-diEPP inhibited the expression and release of IL-6 and IL-10 but no significant change was seen for TNF-α. The results of BMDMs from nAChR gene-deficient mice suggested that nAChR subunit α7 but not α9/α10 plays a role in the signaling of *p*CF3-diEPP. In a similar experiment on human whole blood cultures, the release of the pro-inflammatory cytokines IL-6, TNF-α and IL-1β was reduced by *p*CF3-diEPP, whereas the anti-inflammatory cytokine IL-10 was unaffected. The differences between mouse BMDMs and human whole blood cells may be due to differences between species or due to differences between monocytes and macrophages. However, increasing the n-number of the mouse experiments by at least twofold might show, that the secretion of TNF-α is also reduced by *p*CF3-diEPP. Regarding the secretion of IL-1β, it has been shown before, that monocytes secrete small amounts of IL-1β in response to LPS alone, whereas macrophages need a second danger signal to process pro-IL-1β and release mature IL-1β (Netea et al., 2010; Gaidt et al., 2016).

The observation that PC was ineffective in the experiments on murine BMDMs deserves further discussion. We demonstrated before, that PC efficiently inhibits the ATP-induced release of monocytic IL-1β and that this effect is mediated *via* nAChR subunits α7, α9 and α10 (Hecker et al., 2015; Richter et al., 2016; Zakrzewicz et al., 2017). However, when PC was given at different time points before addition of BzATP, its inhibitory function was time-dependently attenuated, which was not the case for *p*CF3-diEPP (Supplementary Figure 1). We concluded from these results, that PC is degraded or absorbed within minutes in cell culture and did not test its effects on human whole blood cells. This observation underlines the need of a stable anti-inflammatory nAChR agonist such as *p*CF3-diEPP for therapeutic use.

P2X7 receptor stimulation by extracellular ATP is a thoroughly investigated second signal that is of high clinical interest as it mediates trauma- or surgery-induced release of IL-1β, which is a trigger for systemic life-threatening inflammation (Rathinam et al., 2012; Cekic and Linden, 2016; Rathinam and Fitzgerald, 2016; Bortolotti et al., 2018). Up to now, it was unknown if the ATP-induced release of IL-1β is also regulated by PC in macrophages and if *p*CF3-diEPP exerts similar effects. Here, we investigated the effects of PC and *p*CF3-diEPP on the ATP-induced IL-1β release by human monocytic THP-1 cells and THP-1-derived M1-like macrophages. As expected, monocytic and macrophage-like THP-1 cells secreted IL-1β in response to LPS and BzATP. IL-1β secretion was efficiently inhibited by PC and by *p*CF3-diEPP. In line with previously published data (Hecker et al., 2015; Richter et al., 2016, 2020), PC and *p*CF3-diEPP signaled *via* nAChR subunits α7, α9 and/or α10, which was demonstrated by the use of the general nAChR antagonist mecamylamine (Innocent et al., 2008) and the conopeptides [V11L, V16D]ArIB specifically antagonizing nAChR subunit α7 (Whiteaker et al., 2008; Hone et al., 2009, 2010) or RgIA4 that antagonizes nAChRs composed of subunits α9 and α10 (Christensen et al., 2017; Romero et al., 2017; Grau et al., 2018). We conclude that PC and *p*CF3-diEPP exert similar functions at monocytic and macrophage-like cells. However, it should be mentioned that, in contrast to mecamylamine, there was a large variation of the data, when conopeptides were used. Of note, *p*CF3-diEPP also reduces the BzATP-mediated IL-1β release by freshly isolated LPS-primed human PBMCs at a very low IC_50_ value in the femtomolar range. Hence, *p*CF3-diEPP seems to have a higher affinity toward monocytic nAChRs containing subunits α7, α9 and/or α10 compared to classical α7 nAChR agonists.

It should be noted that previous studies indicated that reduction of inflammatory pain could be achieved by inhibition of α9 nAChRs, such that administration of RgIA4 or related analogs was intrinsically analgesic (Christensen et al., 2017; Romero et al., 2017; Huynh et al., 2019; Zheng et al., 2020, 2021). However, the present work shows that the anti-inflammatory activity of PC and *p*CF3-diEPP can be blocked by RgIA4, suggesting that stimulation of α9 nAChRs is anti-inflammatory. Previous *in vivo* studies, however, indicated that a single dose of RgIA4 had a long-lasting effect on neuropathic pain, although it is known, that RgIA4 has a very short half-life *in vivo* (Christensen et al., 2017; Romero et al., 2017). This would suggest that there may be downstream mediators of RgIA4 effects *in vivo*. In contrast, our results indicate that the immediate effect of the conopeptides on the immune cells tested are pro-inflammatory, because they enable the release of pro-inflammatory cytokines in the presence of endogenous cholinergic agonists.

Irrespective of the nAChR agonist used, the nAChR subunit α9 is essential in the cholinergic inhibition of the ionotropic function of the P2X7 receptor. This suggests that *p*CF3-diEPP interacts with α9 nAChRs or α9α10 nAChRs, which turned out to be true. In two-electrode voltage-clamp measurements in *Xenopus laevis* oocytes heterologously expressing human α9 or α9α10 nAChR, *p*CF3-diEPP was a partial agonist that induced about one third of the maximal peak currents stimulated by ACh with a concentration-response relationship resembling that of ACh. This result was unexpected because *p*CF3-diEPP is a poor inducer of ion-currents at α7 nAChR (Quadri et al., 2017).

*p*CF3-diEPP is a silent agonist that favors the desensitized state of α7 nAChRs and, hence, inhibits the ionotropic response to conventional nAChR agonists (Quadri et al., 2017). Here, we investigated if PC exerts similar functions at heterologously expressed human α7, α9 or α9α10 nAChRs. PC had essentially no effect on ACh-induced ion-currents at α7 nAChRs, but it slightly reduced ion-current changes provoked by pulses of ACh on oocytes expressing α9 or α9α10 nAChRs. These mild effects seem to be in contrast to the known strong PC-mediated inhibition of the ionotropic responses to choline (Richter et al., 2016). However, in these previous experiments choline was used instead of ACh (Richter et al., 2016) and the EC_50_ of choline at α9 or α9α10 nAChRs is considerably higher than that of ACh (McIntosh et al., 2009; Boffi et al., 2017; Moglie et al., 2021). PC and conventional nAChR agonists seem to compete for the ligand binding sites at α9 or α9α10 nAChRs and PC functions as a weak to moderate silent agonist or partial antagonist.

The same kind of experiments were performed with 30 μM *p*CF3-diEPP and its known silent agonist function at α7 nAChRs was confirmed. Interestingly, bath application of 30 μM *p*CF3-diEPP also fully inhibited ACh-gated ion-currents at heterologously expressed α9 and α9α10 nAChRs. At the lower concentration of 1 μM, *p*CF3-diEPP was still fully effective at suppressing the transient responses of α9 or α9α10 nAChRs to 60 μM ACh applications, whereas no effects were seen at α7 nAChRs. While being a moderate partial agonist at α9 or α9α10, *p*CF3-diEPP has a higher potential to desensitize these nAChRs compared to α7 nAChRs. It should also be noted that agonistic functions of *p*CF3-diEPP are not detected at concentrations of 1 μM and below, suggesting that *p*CF3-diEPP functions as a desensitizing agent at this low concentration. While the presence of PC or *p*CF3-diEPP in the bath suppressed ACh-evoked responses, upon washout of these compounds the ACh responses, most notably those of the α9/α10 nAChR expressing cells, recovered to levels higher than the initial ACh controls. This suggest that initial ACh responses may have been limited by resting desensitization and that the compounds had the additional effect of altering the equilibrium between pre-desensitized and activatable receptors.

This study has numerous limitations, and without a doubt further studies are needed to characterize the properties of *p*CF3-diEPP in more detail. Different cell types of murine and human origin were investigated, which is of advantage but also a limitation, because not all experiments were performed with all cell types. However, all cell types included in this study express nAChR subunits α7, α9 and α10 (Kawashima et al., 2007; Benfante et al., 2011; Hecker et al., 2015; St-Pierre et al., 2016; Fujii et al., 2017a). The n-number of experiments on BMDMs from gene-deficient animals is too low to draw firm conclusions regarding the regulation of LPS-induced secretion of TNF-α and IL-10, and more nAChR gene-deficient mouse strains need to be tested including those with a single deficiency in nAChR α10 and mice with a triple gene deficiency in nAChR α7α9α10. The concentrations of *p*CF3-diEPP used in this study were based on previous studies, where this compound was characterized as a silent agonist at α7 nAChRs (Quadri et al., 2017). The concentration-response relationship of the *p*CF3-diEPP-mediated control of IL-1β release by human PBMCs and the two-electrode voltage-clamp experiments, in which *p*CF3-diEPP was applied together with ACh in *Xenopus* oocytes, suggest that much lower concentrations of *p*CF3-diEPP are needed to activate metabotropic functions at α9 or α9α10 nAChRs or to desensitize these receptors. More careful analyses are needed to establish the concentration-response relationship for receptor desensitization. Further, more silent agonists of α7 nAChRs have been described (Horenstein and Papke, 2017; Quadri et al., 2017; Godin et al., 2020), which should be tested for their interaction with α9 and α9α10 nAChRs to identify and characterize ideal candidates for preclinical studies. Finally, experiments on LPS-induced cytokine release and BzATP-induced IL-1β release originate from two independent laboratories, which has the disadvantage that different experimental protocols and different murine and human cells were studied. Hence for both experimental settings, further monocytic phagocytes of diverse origin should be investigated. Finally, we did not test if *p*CF3-diEPP induces ion-currents at monocytic phagocytes.

Despite these limitations, *p*CF3-diEPP or related compounds seem to be promising future medicaments These findings raise the possibility of other approaches for the treatment of pain. Positive allosteric modulators (PAMs) that target α7 nAChRs have shown promise (Uteshev et al., 2002; Freitas et al., 2013) as analgesics. Developing PAMs that target receptors containing α9, and/or α10 nAChR subunits, may provide distinct advantages. Chronic use of agonists alone can have the undesired effect of disrupting the endogenous cholinergic tone either through ongoing receptor activation or desensitization. In contrast, PAMs can amplify the effect of endogenous agonists at the therapeutic site or potentiate the effect of exogenously administered agonists. Interestingly, m-bromo PEP, another synthetic silent nAChR agonists based on the diEPP scaffold, protects against EAE (Godin et al., 2020).

Another interesting application might be the prevention of life-threatening trauma-induced inflammation, either as an emergency treatment for accident victims or in the context of major surgery. Although IL-1β is a central pathogenic factor for life-threatening post-operative systemic inflammation that can cause sepsis, therapies targeting the IL-1 system were unsuccessful so far (Bortolotti et al., 2018), most probably because these therapies increase the risk for infectious complications. The *p*CF3-diEPP-mediated control of inflammasome-independent cytokines such as IL-6 or TNF-α seems to be mediated *via* α7 nAChRs and requires relatively high drug concentrations, whereas for the control of the ATP-induced release of IL-1β, the highly *p*CF3-diEPP-sensitive nAChR subunit α9 is mandatory. Low therapeutic concentrations of *p*CF3-diEPP might have the advantage of preventing trauma-induced sterile inflammation, while sparing most pathways that are involved in host-defense against infections.

In conclusion, we provide evidence that *p*CF3-diEPP is a potent agonist at unconventional nAChRs expressed by human monocytic cells. Activation of these receptors can inhibit both, the synthesis of pro-inflammatory cytokines in response to LPS and the inflammasome-dependent maturation and release of IL-1β triggered by P2X7 receptor activation. More research is needed to evaluate the potential of *p*CF3-diEPP to prevent trauma-induced inflammation, inflammatory or autoimmune diseases and neuropathic pain.

## Data Availability Statement

The original contributions presented in the study are included in the article/Supplementary Material, further inquiries can be directed to the corresponding author/s.

## Ethics Statement

The studies involving human participants were reviewed and approved by the Laurentian University Research Ethics Board for Research Involving Human Participants (file number 6012214) and the Ethics Committee of the Medical Faculty Giessen, Giessen, Germany (No. 90/18). The patients/participants provided their written informed consent to participate in this study. The animal study was reviewed and approved by the Boys Town National Research Hospital Institutional Animal Care Committee, Omaha, NE, United States (protocol #16-04), the Laurentian University Animal Care Committee, Sudbury, ON, Canada (file number 6013816) and the University of Florida Institutional Animal Care and Use Committee, Gainesville, FL, United States (approval number 202002669). Written informed consent was obtained from the owners for the participation of their animals in this study.

## Author Contributions

KR participated in the research design, performance of experiments, interpretation of the data, and writing of the manuscript. CS conducted the oocyte experiments, participated in the interpretation of the data, and writing of the manuscript. DCR, ESE, PMKW, and VKS participated in performance of the experiments, interpretation of the data, and editing of the manuscript. AH, JL, WP, K-DS, and MR participated in the research design, interpretation of the data, and editing of the manuscript. ARS, VG, RLP, NAH, JMM, and BJM participated in the research design, interpretation of the data, writing and revision of the manuscript. Additionally, RLP conducted all analyses of the oocyte data, prepared the related figures, and wrote the sections related to the oocyte experiments. All authors contributed to the article and approved the submitted version.

## Conflict of Interest

*p*CF3-diEPP is the subject of a United States provisional patent application, filed in the names of the University of Florida, the University of Giessen, and the Northern Ontario School of Medicine. RLP, CS, NAH, VG, KR, and ARS are listed as inventors on that patent. Certain conopeptides, including RgIA4, have been patented by the University of Utah; JMM is an inventor on these patents. The remaining authors declare that the research was conducted in the absence of any commercial or financial relationships that could be construed as a potential conflict of interest.

## Publisher’s Note

All claims expressed in this article are solely those of the authors and do not necessarily represent those of their affiliated organizations, or those of the publisher, the editors and the reviewers. Any product that may be evaluated in this article, or claim that may be made by its manufacturer, is not guaranteed or endorsed by the publisher.
