# Supplementary material for: Comparison of the Anti-inflammatory Properties of Two Nicotinic Acetylcholine Receptor Ligands, Phosphocholine and pCF3-diEPP
Source: Front Cell Neurosci. 2022 Mar 31;16:779081. doi: 10.3389/fncel.2022.779081 (PMC9008208; doi:10.3389/fncel.2022.779081)
Supplement: Supplementary file 1 [file Data_Sheet_1.pdf]

Supplementary Material

Supplementary Figures

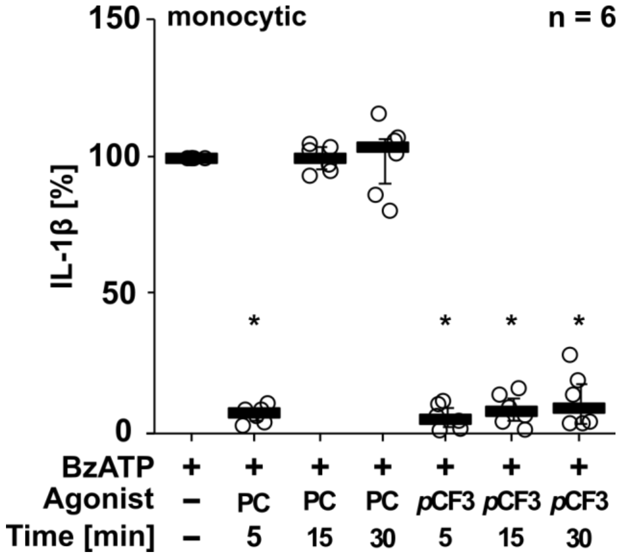

Supplemental Figure S1: Stability of phosphocholine (PC) and *p*CF3-diEPP (*p*CF3) in cell culture experiments. Monocytic THP-1 cells were primed with lipopolysaccharide (LPS; 1 µg/ml, 5 h). Thereafter, the P2X7 receptor agonist BzATP ((2'/3'-O-(4-benzoylbenzoyl)adenosine-5'-triphosphate, tri(triethylammonium) salt) was added for another 40 min to trigger IL-1β release, which was measured by ELISA. To investigate the stability of PC (200 µM) and *p*CF3-diEPP (100 µM) in cell culture, the agonists were added to LPS-primed monocytic THP-1 cells 5, 15 and 30 min before stimulation with BzATP (100 µM). The concentration of IL-1β released in response to BzATP was calculated by subtracting the IL-1β concentrations measured in supernatants of cells treated with LPS alone. In each experiment, the IL-1β concentration obtained after stimulation with BzATP was set to 100% and all other values were calculated accordingly. Data are presented as individual data points, bars represent median, whiskers encompass the 25th to 75th percentile. *$p \leq 0.05$, different from LPS-primed cells stimulated with BzATP alone. Friedman-test followed by the Wilcoxon signed-rank test.

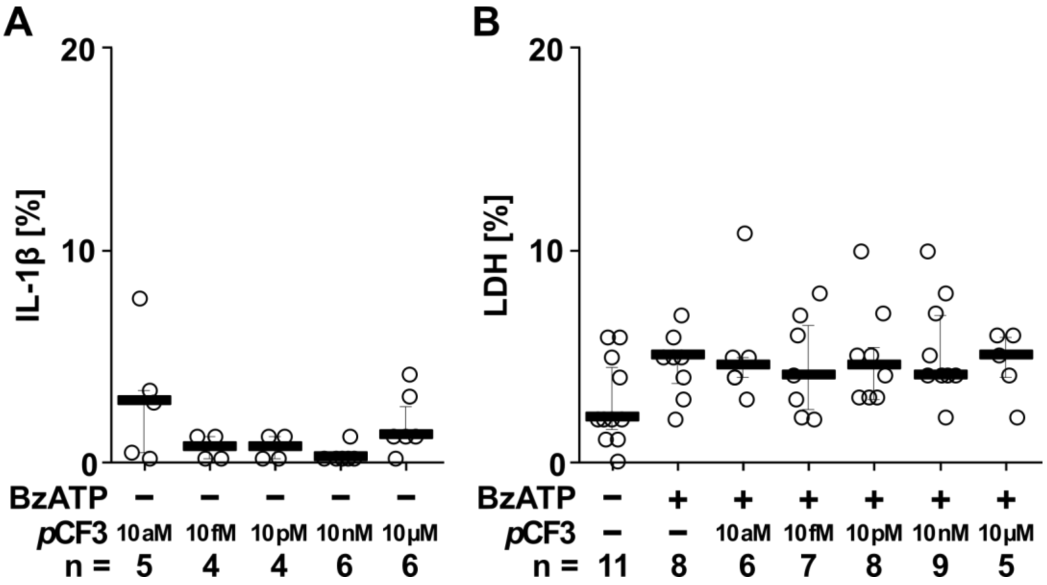

Supplemental Figure S2: Control experiments and test for cell death in human peripheral blood mononuclear cells (PBMCs). PBMCs were freshly isolated from blood of healthy human volunteers and primed with lipopolysaccharide (LPS, 5 ng/ml) during the isolation process. After 3 h of culture, PBMCs were purified by adherence selection. (**A**) The silent agonist *p*CF3-diEPP (*p*CF3) did not induce changes in IL-1β levels of LPS primed human PBMCs in the absence of the P2X7 receptor agonist BzATP ((2’/3’-O-(4-benzoylbenzoyl)adenosine-5’-triphosphate, tri(triethylammonium) salt, 100 µM). (**B**) Cell death was estimated by measurement of lactate dehydrogenase (LDH) in cell free cell culture supernatants. Data are presented as individual data points, bars represent median, whiskers encompass the 25th to 75th percentile.
